# Soluble TREM2 Is Elevated in Pediatric Patients with Anti-NMDAR Encephalitis

**DOI:** 10.3390/jcm15031010

**Published:** 2026-01-27

**Authors:** Anna Zhou, Changhong Ren, Ji Zhou, Xiaotun Ren, Weihua Zhang

**Affiliations:** Department of Neurology, Beijing Children’s Hospital, Capital Medical University, Beijing 100045, China; zhouanna90@sina.com (A.Z.);

**Keywords:** NMDARE, pediatric, TREM2

## Abstract

**Objectives:** Anti-N-methyl-D-aspartate receptor (NMDAR) encephalitis is an autoimmune encephalitis that can lead to severe neurological impairments, particularly in pediatric patients. Effective biomarkers for diagnosis and prognosis are crucial for improved treatment outcomes. To evaluate the potential of soluble Triggering Receptor Expressed on Myeloid cells 2 (sTREM2) in cerebrospinal fluid (CSF) and serum as diagnostic and prognostic biomarkers in pediatric patients with anti-NMDAR encephalitis. **Methods:** The study included 21 children diagnosed with anti-NMDAR encephalitis and 27 children with non-inflammatory neurological disorders (OND) as controls. CSF and serum samples were collected from each patient. sTREM2 levels were measured using enzyme-linked immunosorbent assay (ELISA). Statistical analyses, including Mann–Whitney U test and ROC curve analysis, were performed to assess the diagnostic and prognostic value of sTREM2. **Results:** sTREM2 levels in CSF and serum were significantly higher in children with anti-NMDAR encephalitis compared to the OND group (*p* < 0.001). CSF sTREM2 levels showed a positive correlation with modified Rankin Scale (mRS) scores and a negative correlation with Glasgow Coma Scale (GCS) scores, suggesting an association with disease severity. ROC curve analysis demonstrated that CSF sTREM2 had a high diagnostic accuracy (AUC = 0.887, *p* < 0.001), while serum sTREM2 showed a slightly lower diagnostic accuracy (AUC = 0.848, *p* < 0.001). Patients with better prognoses had significantly lower CSF sTREM2 levels than those with poorer outcomes (*p* = 0.029). **Conclusions:** Elevated CSF sTREM2 levels were associated with increased neuroinflammation and poorer clinical outcomes in children with anti-NMDAR encephalitis. These findings suggest that CSF sTREM2 may serve as a valuable biomarker for the diagnosis and prognosis of pediatric anti-NMDAR encephalitis.

## 1. Introduction

Anti-N-methyl-D-aspartate receptor encephalitis (Anti-NMDAR encephalitis) is an autoimmune encephalitis caused by antibodies generated within the patient that attack the N-methyl-D-aspartate receptors in the brain [1,2,3]. The 2022 Chinese consensus on autoimmune encephalitis identifies six primary manifestations of anti-NMDAR encephalitis: 1. psychiatric or cognitive disturbances; 2. speech dysfunction; 3. seizures; 4. movement disorders or dyskinesia; 5. reduced consciousness; 6. autonomic dysfunction or central hypoventilation [4]. This condition has a relatively low incidence rate of 1.5 per million people [5] and, although it can occur at any age, children and adolescents account for 37% of cases [6]. Treatment for anti-NMDAR encephalitis primarily revolves around immunosuppression and removal of any underlying causes, divided into first-line and second-line therapies [3,7,8,9]. First-line treatments include high-dose corticosteroids (e.g., methylprednisolone), intravenous immunoglobulin (IVIG), plasma exchange, and tumor removal [8,10,11,12,13,14,15,16]. For patients who do not respond to first-line therapies, second-line treatments, such as rituximab and cyclophosphamide, which more aggressively suppress the immune system, are considered [12,16,17,18,19]. Diagnosis of anti-NMDAR encephalitis requires three criteria: 1. the patient must exhibit one or more of the six main clinical manifestations mentioned; 2. positive cerebrospinal fluid (CSF) anti-NMDA receptor antibody test; 3. reasonable exclusion of other diseases [4]. A positive CSF anti-NMDA receptor antibody test is the gold standard for diagnosis [3]. Additionally, other biomarkers such as IL-6, TNF-α, YKL-40, and Fas have been detected at elevated levels in the CSF of patients with anti-NMDA receptor encephalitis [20,21,22,23]. However, these markers lack specificity for the diagnosis of anti-NMDAR encephalitis and their prognostic value remains unclear. Given these limitations, the development of more biomarkers for the diagnosis and prognosis prediction of anti-NMDAR encephalitis is necessary.

Triggering receptor expressed on myeloid cells 2 (TREM2) is an immune receptor expressed on the surface of myeloid cells, predominantly expressed by microglia in the central nervous system and plays a crucial role in the immune-related functions of microglial activation [24,25]. Soluble triggering receptor expressed on myeloid cells 2 (sTREM2) is a fragment generated by the cleavage of the extracellular domain of TREM2 by enzymes such as γ-secretase and ADAM proteases, and can be found in serum and cerebrospinal fluid [26,27,28,29]. Previous studies have indicated that elevated levels of sTREM2 reflect increased microglial activation and exacerbated neuroinflammation [30,31,32,33,34]. In summary, sTREM2 holds significant potential value in the diagnosis of central nervous system diseases, monitoring disease progression, and assessing prognosis.

This study aims to explore the relationship between serum and cerebrospinal fluid (CSF) levels of soluble triggering receptor expressed on myeloid cells 2 (sTREM2) and pediatric anti-NMDAR encephalitis. Additionally, by analyzing the correlation of sTREM2 levels in serum and CSF samples from children with anti-NMDAR encephalitis and a control group with other clinical indicators, this research seeks to unveil the association between sTREM2 levels in serum and CSF and clinical inflammatory markers.

## 2. Methods

### 2.1. Study Design and Biological Sample Collection

This study included 21 pediatric patients diagnosed with anti-NMDAR encephalitis, who were treated at the Department of Neurology, Beijing Children’s Hospital affiliated with Capital Medical University, from March 2022 to September 2023. The diagnosis of anti-NMDAR encephalitis was confirmed in these patients by two physicians according to the revised 2022 diagnostic criteria for anti-NMDAR encephalitis, based on clinical presentations and specific anti-NMDAR antibodies in the cerebrospinal fluid [4]. Exclusion criteria: (1) active or recent infections (bacterial, viral, or fungal) within 2 weeks prior to enrollment, (2) systemic inflammatory diseases (e.g., systemic lupus erythematosus, rheumatoid arthritis), (3) other neuroinflammatory or autoimmune diseases (e.g., multiple sclerosis, sarcoidosis), (4) recent major surgeries or trauma, (5) comorbidities that could significantly impact sTREM2 levels (e.g., chronic kidney disease, liver disease).

Additionally, 27 children with non-inflammatory neurological disorders (ONDs) were selected as the control group. This group included cases of functional headaches (10 cases), vertigo (3 cases), conversion disorder (2 cases), paroxysmal events (1 case), increased intracranial pressure (1 case), Leigh syndrome (1 case), mitochondrial disease (1 case), febrile convulsions (1 case), migraines (1 case), emotional disturbances (1 case), concomitant strabismus (1 case), involuntary movements (1 case), somatization disorder (1 case), congenital external ophthalmoplegia (1 case), and brachial plexus injury (1 case). All participants in the OND group were carefully screened, and none had clinically overt inflammatory etiologies. We recorded the time from the onset of symptoms to the collection of CSF and serum samples, which were obtained within 1 to 14 after symptom onset during the acute phase of the disease. All samples were obtained prior to the initiation of any treatment. Specifically, samples were collected within the acute phase of the disease, before the administration of steroids or intravenous immunoglobulin (IVIG). The timing of treatment initiation, including the dosage of steroids and IVIG, was carefully recorded for each patient. At the time of sample collection, the clinical severity of the patients was assessed using the Glasgow Coma Scale (GCS) and Modified Rankin Scale (mRS) scores. The clinical status of the patients, including their level of consciousness and functional impairment, was also documented to assess the potential impact of treatment initiation timing on biomarker levels. Cerebrospinal fluid and serum samples were collected and immediately centrifuged at 1000× *g* for 10 min. The supernatants were then stored at −80 °C for subsequent ELISA testing. Sample analysis was performed in a blinded manner to minimize potential bias in the measurement of sTREM2 levels.

### 2.2. Clinical Information

This study’s clinical data includes demographic information (age, gender), Glasgow Coma Scale (GCS) scores, and modified Rankin Scale (mRS) scores. These clinical details were sourced from electronic medical records, with GCS and mRS utilized to assess the disease progression and neurological function of the children.

The Glasgow Coma Scale is a clinical tool used to evaluate a patient’s level of consciousness and is widely used in critical care and neuroscience [35]. This scale comprises three components: eye response, verbal response, and motor response. The total score, which ranges from 3 to 15, is the sum of these three components. Scores from 3 to 8 indicate severe consciousness impairment, 9 to 12 suggest moderate consciousness impairment, and 13 to 15 indicate mild consciousness impairment or normalcy.

The modified Rankin Scale is utilized to assess the functional status of patients following the acute phase of a neurological disorder, measuring the extent of their functional recovery and severity of disability [36]. This scale’s scoring system ranges from 0 to 6, across seven levels: no symptoms, no significant disability, slight disability, moderate disability, moderately severe disability, severe disability, and death. In this study, mRS scores were assessed through face-to-face structured interviews.

### 2.3. Ethical Approval and Informed Consent

This experiment was conducted in accordance with the ethical principles and guidelines set forth in the Declaration of Helsinki. The children and their families were informed about their participation in this study and provided written informed consent. This study was approved by the Ethics Committee of Beijing Children’s Hospital affiliated with Capital Medical University (Ethics Approval Number: IEC-C-006-A04-V.06).

### 2.4. ELISA Detection of sTREM2

In this study, we quantified the levels of soluble Triggering Receptor Expressed on Myeloid cells 2 (sTREM2) in serum and cerebrospinal fluid using an enzyme-linked immunosorbent assay (ELISA) kit (product number ab224881, Abcam, Cambridge, UK). All assays were meticulously performed according to the manufacturer’s protocol provided in the kit manual. To ensure accuracy and reproducibility, each standard and sample was assayed in duplicate.

### 2.5. Statistical Analysis

Statistical analyses in this study were performed using SPSS version 24.0 (IBM, Armonk, NY, USA) and GraphPad Prism version 8.0. Quantitative data are presented as mean ± standard deviation (SD) or median with interquartile range, depending on the results of normality testing. Differences in cerebrospinal fluid (CSF) white blood cell (WBC) counts, and serum and CSF sTREM2 levels between the anti-NMDAR encephalitis group and the control group were assessed using the Mann–Whitney U test. Gender disparities between the anti-NMDAR encephalitis group and the OND (other non-inflammatory disorders) control group were evaluated using Yates’ corrected chi-square test. Pearson’s two-tailed correlation test was utilized to compute correlation coefficients for variables adhering to a normal distribution. For variables not following a normal distribution, Spearman’s two-tailed correlation test was applied. The diagnostic utility was gauged through receiver operating characteristic (ROC) curves. A *p*-value of less than 0.05 was deemed to indicate statistical significance.

## 3. Results

### 3.1. Patient Characteristics

The baseline clinical information and sTREM2 levels for the anti-NMDAR encephalitis group (*n* = 21) and the OND control group (*n* = 27) are detailed in Supplementary Materials. All children in the OND group tested negative for specific NMDAR antibodies in cerebrospinal fluid (CSF). The age of children in the anti-NMDAR encephalitis group was slightly lower compared to the OND group, with mean ages of 114.3 ± 41.66 months and 103.6 ± 60.53 months, respectively, although this difference was not statistically significant (*p* = 0.075). There was no statistically significant difference in the gender distribution between the two groups.

The cerebrospinal fluid (CSF) white blood cell (WBC) count was significantly higher in the anti-NMDAR encephalitis group compared to the OND group, suggesting a heightened level of neuroinflammation associated with anti-NMDAR encephalitis. Additionally, Glasgow Coma Scale (GCS) scores indicated that within the anti-NMDAR encephalitis group, 11 children scored between 13 and 15, 6 scored between 9 and 12, and 4 scored between 3 and 8. Modified Rankin Scale (mRS) scores showed that 9 children were assessed with a score of 5, 6 with a score of 4, 4 with a score of 3, 1 with a score of 2, and 1 with a score of 1 (Supplementary Materials). These results from both scales suggest that nearly half of the children in the acute phase of anti-NMDAR encephalitis exhibited moderate to severe consciousness impairments. Furthermore, the majority of these children displayed significant functional disabilities, directly affecting their daily living capabilities, reflecting the severe impact of anti-NMDAR encephalitis on affected children during its acute phase.

The CSF white blood cell (WBC) count in the anti-NMDAR encephalitis group was significantly higher than that in the OND group, with counts of 35.0 (5.0–49.0) × 10^6^/L compared to 6.0 (2.0–9.0) × 10^6^/L in the OND group (*p* = 0.005). This significant increase indicates a higher level of neuroinflammation in the anti-NMDAR encephalitis group. Regarding CSF total protein concentrations, there was no significant difference between the groups, with the OND group at 233.0 (189.0–320.0) ng/mL and the anti-NMDAR group at 286.0 (191.5–373.5) ng/mL (*p* = 0.276) (Supplementary Materials).

### 3.2. Elevated sTREM2 Levels in Serum and Cerebrospinal Fluid of Children with Anti-NMDAR Encephalitis

**Figure 1:**
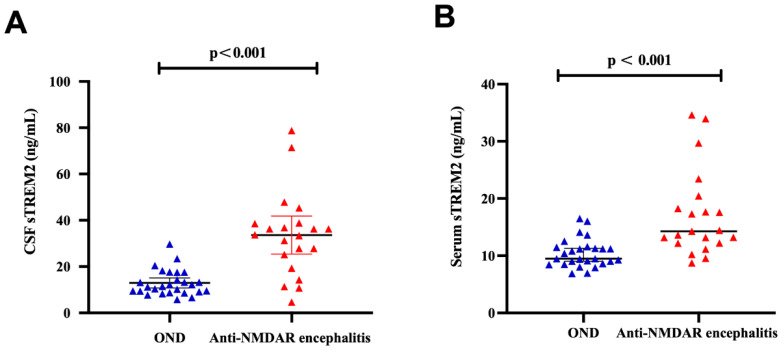
**High level of CSF and serum sTREM2 in anti-NMDAR encephalitis.** (**A**) sTREM2 levels in cerebrospinal fluid samples from the Anti-NMDAR encephalitis group (*n* = 21) were significantly higher than the OND groups (*n* = 27) (*p* < 0.001). (**B**) sTREM2 levels in serum samples from the Anti-NMDAR encephalitis group (*n* = 21) were significantly higher, compared with the OND group (*n* = 27) (*p* < 0.001). The differences in CSF levels of sTREM2 were analyzed using an independent two-sample *t*-test, while the differences in serum sTREM2 were assessed using the Mann–Whitney U test.

The levels of soluble triggering receptor expressed on myeloid cells 2 (sTREM2) in the cerebrospinal fluid (CSF) of the anti-NMDAR encephalitis group were significantly higher, averaging 33.64 ± 17.99 ng/mL, compared to 12.22 ± 5.68 ng/mL in the OND group. Additionally, the median serum sTREM2 levels in the anti-NMDAR encephalitis group were 14.28 ng/mL (range: 12.21–19.37 ng/mL), whereas in the OND group, the levels were 9.48 ng/mL (range: 8.64–11.49 ng/mL). Both CSF and serum sTREM2 levels were significantly elevated in the anti-NMDAR encephalitis group compared to the OND group (*p* < 0.001) as shown in Figure 1A,B.

### 3.3. Correlation of sTREM2 Levels with Clinical Parameters in Children with Anti-NMDAR Encephalitis

**Figure 2:**
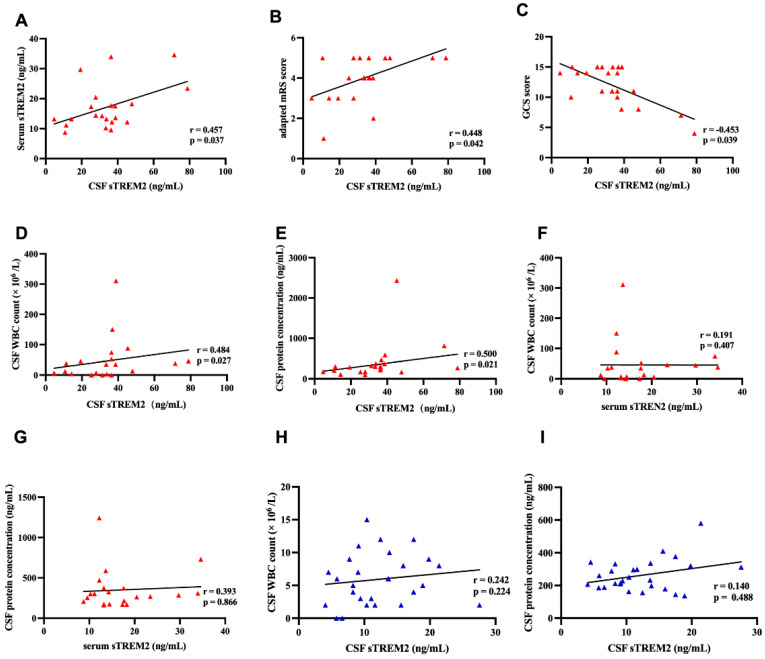
**The CSF sTREM2 levels were associated with mRS scores, GCS scores, and clinical inflammatory indicators.** (**A**) In the Anti-NMDAR encephalitis group, CSF sTREM2 levels were significantly positively correlated with serum sTREM2 levels (Pearson correlation, r = 0.457, *p* = 0.037). (**B**) There was a positive correlation between the modified Rankin Scale (mRS) scores and CSF sTREM2 levels (Spearman correlation, r = 0.448, *p* = 0.042). (**C**) CSF sTREM2 levels were negatively correlated with Glasgow Coma Scale (GCS) scores (Spearman correlation, r = −0.453, *p* = 0.039). (**D**,**E**) Significant correlations were found between CSF sTREM2 levels and central clinical inflammatory indicators. CSF sTREM2 levels were significantly positively correlated with CSF WBC counts (Spearman correlation, r = 0.484, *p* = 0.027). CSF sTREM2 levels were also significantly positively correlated with CSF protein concentrations (Spearman correlation, r = 0.500, *p* = 0.021). (**E**) CSF sTREM2 levels were negatively correlated with Glasgow Coma Scale (GCS) scores (Spearman correlation, r = −0.453, *p* = 0.039). (**F**,**G**) In the Anti-NMDAR encephalitis group, there was no correlation between CSF sTREM2 levels and CSF WBC counts (Spearman correlation, r = 0.191, *p* = 0.407) or CSF protein concentrations (Spearman correlation, r = 0.393, *p* = 0.866). (**H**,**I**) In the OND group, there was no correlation between serum sTREM2 levels and CSF WBC counts (Spearman correlation, r = 0.242, *p* = 0.224) or CSF protein concentrations (Spearman correlation, r = 0.140, *p* = 0.488).

In children diagnosed with anti-NMDAR encephalitis, the levels of soluble Triggering Receptor Expressed on Myeloid cells 2 (sTREM2) in cerebrospinal fluid (CSF) correlate with clinical neurological function scores and inflammation markers. Demonstrated in Figure 2, there is a statistically significant positive correlation between CSF sTREM2 levels and serum sTREM2 levels within the anti-NMDAR encephalitis group (r = 0.457, *p* = 0.037) (Figure 2A). Furthermore, CSF sTREM2 levels are positively correlated with the modified Rankin Scale (mRS) scores of these children (r = 0.448, *p* = 0.042) (Figure 2B) and show a negative correlation with the Glasgow Coma Scale (GCS) scores (r = −0.453, *p* = 0.039) (Figure 2C).

In terms of inflammation, CSF sTREM2 levels show a positive correlation with both CSF white blood cell count (r = 0.484, *p* = 0.027) and CSF protein concentration (r = 0.500, *p* = 0.021) (Figure 2D,E). However, the correlations between serum sTREM2 levels and both CSF white blood cell count (r = 0.191, *p* = 0.407) and CSF protein concentration (r = 0.393, *p* = 0.866) in the anti-NMDAR encephalitis group are not significant (Figure 2F,G).

Furthermore, in the OND control group, the correlations between CSF sTREM2 levels and both CSF white blood cell count (r = 0.242, *p* = 0.224) and CSF protein concentration (r = 0.140, *p* = 0.488) are also not significant (Figure 2H,I). This suggests that sTREM2 may specifically correlate with the severity and inflammatory state in anti-NMDAR encephalitis but not in other non-inflammatory neurological disorders.

As shown in Supplementary Materials (Table S3), we present the correlation coefficients (r) and corresponding *p*-values for CSF and serum sTREM2 levels with various clinical and laboratory parameters.

### 3.4. CSF sTREM2 Levels as a Potential Prognostic Marker in Anti-NMDAR Encephalitis

**Figure 3:**
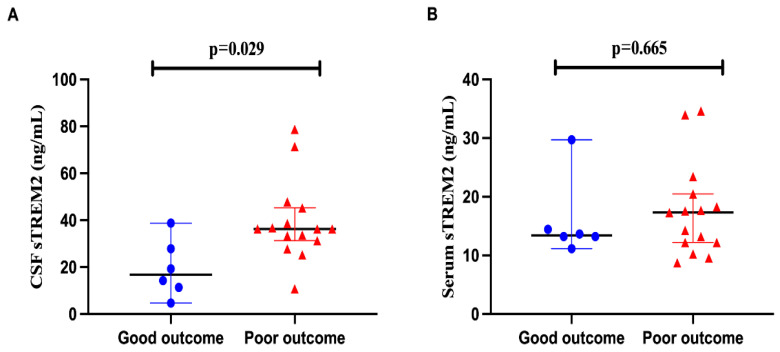
**The CSF sTREM2 level has the potential to assess the prognosis of anti-NMDAR encephalitis.** (**A**,**B**) Each patient’s clinical outcome was classified as good or poor according to the mRS score (mRS  >  3 poor outcome, mRS  ≤  3 good outcome). In anti-NMDAR encephalitis, CSF sTREM2 showed lower concentrations in patients with good outcomes than in patients with poor outcomes (*p* = 0.029). In contrast, serum sTREM2 level showed no significant difference between the two outcomes (*p* = 0.665).

In assessing the prognostic utility of cerebrospinal fluid (CSF) soluble Triggering Receptor Expressed on Myeloid cells 2 (sTREM2) levels in anti-NMDAR encephalitis, clinical outcomes were delineated by the modified Rankin Scale (mRS) scores for the children involved. Outcomes were categorized as either poor (>3) or good (≤3). In patients with anti-NMDAR encephalitis, those with a favorable prognosis demonstrated significantly lower CSF sTREM2 levels compared to those with an unfavorable prognosis (*p* = 0.029). However, the differences in serum sTREM2 levels between these two outcome groups were not statistically significant (*p* = 0.665), as depicted in Figure 3A,B.

### 3.5. CSF and Serum sTREM2 as Biomarkers for Diagnosing Anti-NMDAR Encephalitis

**Figure 4:**
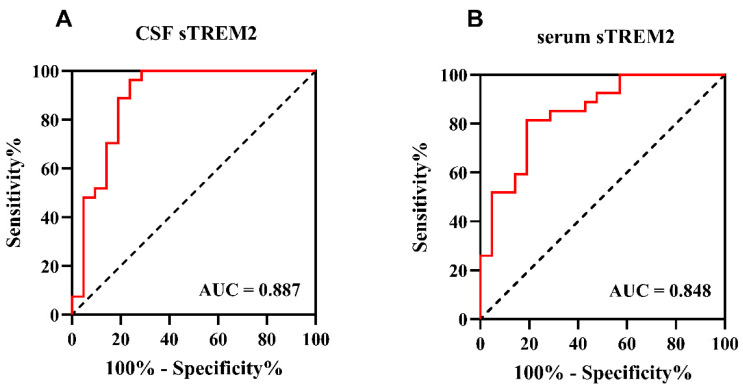
**The CSF and serum sTREM2 levels can be a specific anti-NMDAR encephalitis diagnosis biomarker.** (**A**,**B**) Receiver operating characteristic (ROC) curve analysis of sTREM2 in CSF and serum for anti-NMDAR encephalitis diagnosis (*n* = 48). (**A**) The area under the curve (AUC) of CSF sTREM2 levels was 0.887 (*p* < 0.001), 95% confidence intervals were 0.780–0.994, cut-off value was 0.725, sensitivity was 76.19%, and specificity was 96.30%. (**B**) The area under the curve (AUC) of serum sTREM2 levels was 0.848 (*p* < 0.001), 95% confidence intervals were 0.739–0.958, cut-off value was 0.625, sensitivity was 81.00%, and specificity was 81.50%.

To further explore the capability of sTREM2 to differentiate children with anti-NMDAR encephalitis from those in the OND group, receiver operating characteristic (ROC) curve analysis was conducted in this study. The results demonstrated that cerebrospinal fluid (CSF) sTREM2 levels could effectively distinguish between children with anti-NMDAR encephalitis (*n* = 21) and those with other non-inflammatory disorders (OND) (*n* = 21), with an area under the curve (AUC) of 0.887 (*p* < 0.001). The discriminatory ability of serum sTREM2 levels was slightly weaker than that of CSF sTREM2, with an AUC of 0.848 (*p* < 0.001), as shown in Figure 4A,B. This indicates that both CSF and serum levels of sTREM2 hold potential as diagnostic biomarkers for anti-NMDAR encephalitis, albeit with CSF levels showing a stronger association.

## 4. Discussion

This study examined soluble Triggering Receptor Expressed on Myeloid cells 2 (sTREM2) levels in cerebrospinal fluid (CSF) and serum of children with anti-NMDAR encephalitis versus non-inflammatory neurological disorders, integrating CSF inflammatory markers (white blood cell count, protein concentration) to explore their relationships. Key findings: (1) CSF and serum sTREM2 were significantly higher in anti-NMDAR encephalitis patients than controls; (2) In the encephalitis group, CSF sTREM2 positively correlated with CSF white blood cell count and protein concentration; (3) CSF sTREM2 positively associated with modified Rankin Scale (mRS) scores and inversely with Glasgow Coma Scale (GCS) scores; (4) CSF sTREM2 was lower in patients with better prognosis; (5) Both CSF and serum sTREM2 could serve as diagnostic biomarkers for anti-NMDAR encephalitis. Notably, serum sTREM2 showed no significant correlation with CSF inflammatory markers, indicating CSF sTREM2 is more effective in characterizing central nervous system inflammation extent. To minimize confounding, participants with active infections (known to elevate sTREM2 via microglial activation and neuroinflammation) were excluded. Given infections are common triggers/complications of anti-NMDAR encephalitis, this exclusion enhanced the validity of our findings.

In the central nervous system (CNS), microglia are the sole expressors of the transmembrane innate immune receptor TREM2 (Triggering Receptor Expressed on Myeloid cells 2). Ligand binding activates TREM2, initiating intracellular signaling cascades that enhance microglial survival, proliferation, chemotaxis, and phagocytosis [37,38,39,40,41]. External stimuli induce TREM2 cleavage and release from the cell surface, forming soluble TREM2 (sTREM2) [42]. Current research identifies CSF sTREM2 as a biomarker of CNS microglial activation [24]. Notably, CSF and serum sTREM2 differ in utility: CSF sTREM2 is a more reliable marker for CNS-specific inflammation (less affected by systemic inflammation), with elevated levels strongly linked to microglial activation and blood–brain barrier dysfunction, especially in acute neuroinflammatory diseases like encephalitis. In contrast, serum sTREM2 reflects broader systemic inflammation, limiting its specificity for CNS pathology. While sTREM2 is a key neuroinflammation biomarker, it is not disease-specific and may indicate a general inflammatory process rather than a specific disorder.

Extensive studies have measured CSF and serum sTREM2 levels in diverse neurological disorders. In neurodegenerative diseases, cross-sectional analyses show Alzheimer’s disease patients have moderately elevated CSF sTREM2 vs. cognitively normal controls [43,44,45]. This increase emerges early in the disease course, correlates strongly with tau pathology and neurodegenerative changes [46,47,48], and has prognostic value: Ewers et al. found higher baseline CSF sTREM2 is linked to slower memory and cognitive decline [43], while Gispert et al. reported that it correlates with greater gray matter volume [49]. These findings imply elevated sTREM2 may protect the brain, highlighting its potential therapeutic significance for neurodegenerative diseases.

The heterogeneous OND control group may confound study results, particularly CSF/serum sTREM2 levels. Enrolled patients had non-inflammatory neurological disorders (e.g., headaches, conversion disorders, mitochondrial diseases); some subtypes (e.g., mitochondrial dysfunction) involve mild neuroinflammation that could alter microglial activation and sTREM2 release. While sTREM2 is elevated in neuroinflammatory states, the impact of specific OND subtypes remains unclear—mitochondrial diseases and paroxysmal events may induce subtle subclinical neuroinflammation affecting sTREM2 levels, even without autoimmune pathology, though supporting data are scarce. The heterogeneity of the control cohort may impose certain limitations on the interpretation of the diagnostic accuracy derived from ROC analyses. Future research with homogeneous controls (minimal neuroinflammation) and OND subgroup analyses will clarify neuroinflammation’s role in sTREM2 regulation among non-autoimmune disorders. Notably, sTREM2 reflects a universal inflammatory response rather than an NMDARE-specific feature.

Elevated CSF sTREM2 levels—linked to better cognitive function in Parkinson’s disease (PD) patients vs. healthy controls [50,51]—correlated with poorer neurological scores in this study; this discrepancy likely reflects the chronicity of diseases in prior research versus the acute pediatric anti-NMDAR encephalitis and shorter timeframe here. Significantly higher CSF sTREM2 levels are also seen in inflammatory neurological disorders (e.g., multiple sclerosis, viral meningitis) than non-inflammatory ones [34], with levels correlating with multiple sclerosis duration [52], indicating sTREM2 is a marker of central nervous system microglial activation rather than a disease-specific indicator. Few studies have explored sTREM2 in anti-NMDAR encephalitis, but Chang et al. reported elevated CSF sTREM2 in adult patients (vs. non-inflammatory controls), correlating with inflammatory markers, neurological scores, and showing diagnostic value [53]—consistent with this study’s pediatric findings. This is the first study to examine CSF and serum sTREM2 in pediatric anti-NMDAR encephalitis, clarifying the link between CSF sTREM2 and clinical neuroinflammatory markers.

## 5. Conclusions

This study presents several limitations. Firstly, the low incidence of the disease limits the sample size (*n* = 21), which may affect the generalizability and statistical power of the findings. The moderate correlations observed between CSF sTREM2 levels and clinical parameters (such as GCS, mRS, and inflammatory markers) lie near the threshold of statistical significance (r ≈ 0.44–0.50), and given the small sample size, there is a high risk of instability in these correlation coefficients. Therefore, we consider these findings to be preliminary, and we emphasize that these results should not be used for clinical stratification or decision-making without external validation. These findings should be viewed as hypothesis-generating rather than conclusive. Future studies with larger sample sizes and multi-center approaches are needed to strengthen the results. Secondly, CSF and serum samples were collected within 1–3 days after the onset of symptoms. While this timeframe captures the acute phase of the disease, the timing of sample collection could influence the levels of sTREM2, as neuroinflammatory markers can fluctuate at different stages of the disease. Thirdly, the cross-sectional nature of this study precludes the direct determination of causality due to the absence of longitudinal data. This study involved a single time-point sample collection, and no repeated or serial samples were obtained from any participants. Future studies with longitudinal sampling could offer additional insights into the temporal dynamics of sTREM2 levels throughout the course of the disease. Finally, this study did not consider a broader spectrum of clinical neuroinflammatory markers, which may restrict our understanding of sTREM2 levels and their interrelations with other potential biomarkers within the scope of neurological inflammation. These limitations underscore the need for further research to validate the findings and explore the broader implications of sTREM2 as a biomarker in anti-NMDAR encephalitis.

## Data Availability

The original contributions presented in this study are included in the article/Supplementary Material. Further inquiries can be directed to the corresponding author(s).

## Supplementary Materials

The following supporting information can be downloaded at: https://www.mdpi.com/article/10.3390/jcm15031010/s1, Table S1: Clinical and demographic characteristics of participants; Table S2: The laboratory parameters of participants; Table S3: Correlation data of of CSF and Serum sTREM2 with clinical and laboratory parameters in two groups.
