# Supplementary material for: Soluble TREM2 Is Elevated in Pediatric Patients with Anti-NMDAR Encephalitis
Source: J Clin Med. 2026 Jan 27;15(3):1010. doi: 10.3390/jcm15031010 (PMC12897725; doi:10.3390/jcm15031010)
Supplement: Supplementary file 1 [file jcm-15-01010-s001.zip › jcm-3966249-supplementary.pdf]

Table S1. Clinical and demographic characteristics of participants.

Subject details	OND (*n*=27)	Anti-NMDAR encephalitis（*n*=21）	t/U/χ²	*P*
Age，M±SD，m	114.3±41.66	103.6±60.53	0.729	0.075
Gender, no. (%)			0.701	0.403
Male	16 (59.26%)	9 (42.86%)		
Female	11 (40.74%）	12（57.14%）		
Glasgow Coma Scale (GCS)				
GCS 13–15, mild head injury	-	11 (52.38)		
GCS 9–12, moderate head injury	-	6 (28.57%)		
GCS 3–8, severe head injury	-	4 (19.05%)		
Modified Rankin Scale (mRS)				
Score 0, no symptoms	-	0		
Score 1, non-disabling symptoms	-	1 (4.76%)		
Score 2, minor symptoms	-	1 (4.76%)		
Score 3, moderate symptoms	-	4 (19.05%)		
Score 4, moderately severe symptoms	-	6 (28.57%)		
Score 5, severely disabled	-	9 (42.86%)		
Score 6, dead	-	0		

Data expressed as mean ± SD or median ± SD (IQR) as appropriate. m, month;

Table S2 The laboratory parameters **of participants.**

Subject details	OND (*n*=27)	Anti-NMDAR encephalitis（*n*=21）	t/U/χ²	*P*
CSF WBC count (× 10^6 /L), median (IQR)	6.0(2.0−9.0)	35.0 (5.0−49.0)	149.5	0.005
CSF protein concentration (ng/mL), median (IQR)	233.0(189.0−320.0)	286.0 (191.5−373.5)	230.5	0.276
Serum sTREM2(ng/mL), median (IQR)	9.48 (8.64−11.49)	14.28 (12.21−19.37)	86	<0.001
CSF sTREM2(ng/mL), M±SD	12.22±5.68	33.64±17.99	5.841	<0.001

Data expressed as mean ± SD or median ± SD (IQR) as appropriate. WBC, white blood cell; CSF, cerebrospinal fluid; sTREM2, soluble triggering receptor expressed on myeloid cell 2.

Table S3. Correlation data of of CSF and Serum sTREM2 with clinical and laboratory parameters in two groups

Group	Variables	Correlation (r)	p-value
Anti-NMDAR encephalitis（n=21）	CSF sTREM2 vs Serum sTREM2	0.457	0.037
	CSF sTREM2 vs Adapted mRS score	0.448	0.042
	CSF sTREM2 vs GCS score	-0.453	0.039
	CSF sTREM2 vs CSF WBC count	0.484	0.027

Group	Variables	Correlation (r)	p-value
	CSF sTREM2 vs CSF protein concentration	0.500	0.021
	Serum sTREM2 vs CSF WBC count	0.191	0.407
	Serum sTREM2 vs CSF protein concentration	0.393	0.866
OND (n=27)	CSF sTREM2 vs CSF WBC count	0.242	0.224
	CSF sTREM2 vs CSF protein concentration	0.140	0.488
